# PROTOCOL: Social interventions to improve well‐being of people with mental disorders: Global evidence and gap map

**DOI:** 10.1002/cl2.1182

**Published:** 2021-07-21

**Authors:** Sherize M. Dsouza, Ashrita Saran, Jisha B. Krishnan

**Affiliations:** ^1^ Department of Public Health, Prasanna School of Public Health Manipal Academy of Higher Education Udupi India; ^2^ Campbell Collaboration Delhi India; ^3^ Public Health Evidence South Asia (PHESA), Prasanna School of Public Health Manipal Academy of Higher Education Manipal India

**Keywords:** mental disorders, mental illness, social Interventions, well‐being

## Abstract

**Introduction**: Mental illnesses play a role in poor health outcomes. Mental health is just as vital as physical health for an individual's total well‐being. Alterations in mental health can have a significant impact on all aspects of life, including school or work performance, relationships with family and friends, and community participation. As a result, we would like to provide an overview of psychosocial interventions that are available to improve the well‐being of people with mental health conditions and map available studies on the effectiveness of interventions provided in framework.

**Methods**: This Evidence Gap Map will feature systematic reviews of the effects of interventions and effectiveness studies that used either: (a) randomised experimental design, or (b) rigorous quasi‐experimental design, (c) natural experiments, (d) regression discontinuity, (e) propensity score matching, (f) difference in difference, (g) instrumental variables, (h) and other matching design, (I) Single subject design. We will include qualitative studies, relevant working papers will also be included.

Also, language restricted to english from any country will be reviewed for inclusion. Electronic Search will be conducted with the help of a relevant databases in our area of study.

**Outcomes**: Will be focused mainly on the basis of community‐based Rehabilitation matrix adapted from the comprehensive mental health action plan, 2013‐2020.

## 1 BACKGROUND

### 1.1 Description of the condition

“Mental health” is defined as a state of wellbeing in which every individual realises his or her own potential, can cope with the normal stresses of life, can work productively and fruitfully, and is able to make a contribution to her or his community (WHO, 2019b).

Special Initiative by the World Health Organisation has been undertaken from the year 2019 to 2023 that seeks to ensure universal health coverage involving access to quality and affordable care for mental health conditions in 12 countries to 100 million more people. The initiative will advance policies, advocacy and human rights, and scale‐up quality interventions and services for people with mental health conditions (Ghebreyesus, 2019).

#### 1.1.1 **G**lobal mental health statistics

Poor mental health conditions being one of the top five contributors to health burden in the year 2010, accounted for about 7.4% in low‐ and middle‐income countries (LMICs) (Herrman & Jané‐Llopis, 2012).

Currently, about 450 million people worldwide have experienced any one/several kind of mental health conditions and behavioural disorders. One in four individuals developed one or more of these mental health conditions during their lifetime (WHO—Effective interventions and policy options, 2019a).

Between 76% and 85% of people with severe mental health conditions receive no treatment in LMICs; the corresponding range for high income countries (HICs) is also high: between 35% and 50%. Neuropsychiatric conditions account for 13% of the total Disability Adjusted Life Years (DALYs) lost due to all diseases and injuries in the world and are estimated to increase to 15% by the year 2020. DALYs for a disease are the sum of the years of life lost due to premature mortality (YLL) in the population and the years lost due to disability (YLD) for incident cases of the health condition. The DALY is a health gap measure that extends the concept of potential years of life lost due to premature death (PYLL) to include equivalent years of “healthy” life lost in states of less than full health, broadly termed disability. Five of the 10 leading causes of disability and premature death worldwide are due to mental ill‐health. Mental illness not only represent an immense psychological, social and economic burden to society, but also increase the risk of physical illnesses (WHO, 2004).

#### 1.1.2 Global evidence

Global target from WHO advert 80% of countries will have developed or updated their policies/plans for mental health in line with international and regional human rights instruments by the year 2020 (SDG‐Mental‐healthupdate‐2018, WHO, 2018).

The disability caused by mental and neurological disorders is high in all regions of the world. As a proportion of the total, however, it is comparatively less in the developing countries, mainly because of the large burden of communicable, maternal, perinatal and nutritional conditions in those regions. Even so, neuropsychiatric disorders cause 17.6% of all YLDs in Africa (Leonardi, 2003).

Taken together, mental, neurological and substance use disorders exact a high toll, accounting for 13% of the total global burden of disease in the year 2004. Depression alone accounts for 4.3% of the global burden of disease and is among the largest single causes of disability worldwide (11% of all years lived with disability globally), particularly for women. The economic consequences of these health losses are equally large: a recent study estimated that the cumulative global impact of mental disorders in terms of lost economic output will amount to US$ 16.3 million between 2011 and 2030 (Comprehensive mental health action plan report, 2013‐2020, WHO, 2013).

#### 1.1.3 Comprehensive mental health action plan 2020, WHO

The vision of the action plan is a world in which mental health is valued, promoted and protected, mental disorders are prevented and persons affected by these disorders are able to exercise the full range of human rights and to access high quality, culturally appropriate health and social care in a timely way to promote recovery, all to attain the highest possible level of health and participate fully in society and at work free from stigmatisation and discrimination. Its overall goal is to promote mental well‐being, prevent mental disorders, provide care, enhance recovery, promote human rights and reduce the mortality, morbidity and disability for persons with mental disorders.

The action plan has the following objectives:

(1) To strengthen effective leadership and governance for mental health;
(2) To provide comprehensive, integrated and responsive mental health and social care services in community‐based settings;
(3) To implement strategies for promotion and prevention in mental health;
(4) To strengthen information systems, evidence and research for mental health.

The global targets established for each objective provide the basis for measurable collective action and achievement by Member States towards global goals and should not negate the setting of more ambitious national targets, particularly for those countries that have already reached global ones.

The action plan relies on six cross‐cutting principles and approaches: but important one for us being the “Evidence‐based practice” that concentrates on mental health strategies and interventions for treatment, prevention and promotion that needs to be based on scientific evidence and/or best practices, taking cultural considerations also into account.

### 1.2 Description of the intervention

#### 1.2.1 Mental health interventions

The WHO Department's work on mental health in emergencies focuses mostly on resource‐poor countries, where most populations exposed to natural disasters and war live.

Some of the interventions have successfully been implemented by community health workers in low‐income countries as part of research programmes that ensured that community health workers had the time to learn and implement these interventions under supervision.

The interventions described below cover both psychological and social interventions (broader categories) that will be included to map in this review.

1. **Behavioural activation**: A component of cognitive behavioural therapy (CBT) for depression, is a psychological treatment that focuses on activity scheduling to encourage a person to stop avoiding activities that are rewarding. The mhGAP‐IG recommends it as a treatment option for depression (including bipolar depression) and other significant emotional or medically unexplained complaints.
2. **CBT**: This therapy is based on the idea that feelings are affected by thinking and behaviour. People with mental disorder tend to have unrealistic distorted thoughts, which if unchecked can lead to unhelpful behaviour. CBT typically has a cognitive component and a behavioural component. CBT may vary according to the mental health conditions. The mhGAP‐IG recommends it as a treatment option for depression (including bipolar depression), behavioural disorders, alcohol use disorders or drug use disorders, and also recommends it as a treatment option for psychosis just after the acute phase.
3. **Family counselling or therapy**: It entails multiple (usually more than six) planned sessions over a period of months. It should be delivered to individual families or groups of families. It has supportive and educational or treatment functions. It often includes negotiated problem‐solving or crisis management work. The mhGAP‐IG recommends it as a therapy for people with psychosis, alcohol use disorders or drug use disorders.
4. **Interpersonal psychotherapy** is a psychological treatment designed to help a person identify and address problems in their relationships with family, friends, partners and other people. The mhGAP‐IG recommends it as a treatment option for depression, including bipolar depression.
5. **Parent skills training for parents of children and adolescents with behavioural disorders**
  Parent skills training for parents of children with behavioural disorders involves training focusing on positive parent–child interactions and emotional communication, teaching the importance of parenting consistency, discouraging harsh punishments and requiring the practice of new skills with their children during the training. Although the content should be culturally sensitive, it should not allow violation of children's basic human rights according to internationally endorsed principles. Providing parent training requires that the health‐care providers receive training themselves.
6. **Problem‐solving counselling or therapy**: A psychological treatment involving offering direct and practical support. The therapist and person work together to identify and isolate key problem areas that might be contributing to the person's mental health problems, to break these down into specific, manageable tasks, and to problem‐solve and develop coping strategies for particular problems. The mhGAP‐IG recommends it as an adjunct treatment option for depression (including bipolar depression) and as a treatment option for alcohol use disorders or drug use disorders. It is also recommended for self‐harm, other significant emotional or medically unexplained complaints, or parents of children and adolescents with behavioural disorders.
7. **Relaxation training**: This intervention involves training the person in techniques such as breathing exercises and progressive relaxation to elicit the relaxation response. Progressive relaxation teaches how to identify and relax specific muscle groups. Usually treatment consists of daily relaxation exercises for at least 1–2 months. The mhGAPIG recommends it as an adjunct treatment option for depression (including bipolar depression), and as a treatment option for other significant emotional or medically unexplained complaints.
8. **Social skills therapy**
  Social skills therapy helps rebuild skills and coping in social situations to reduce distress in everyday life. It uses role‐playing, social tasks, encouragement and positive social reinforcement to help improve ability in communication and social interactions. Skills training can be done with individuals, families and groups. Usually treatment consists of 45–90 min sessions once or twice per week for an initial 3 months and then every month. The mhGAP‐IG recommends it as a treatment option for people with psychosis or behavioural disorder.

A new WHO *self‐help approach* for managing distress and coping with adversity has shown to be safe and effective in a trial involving South Sudanese women living in Uganda. The results of the study indicate that guided self‐help could be a promising strategy to address the vast gap in mental health support in humanitarian response situations (Brown et al., 2018).

Hence, sound mental health is related to mental and psychological well‐being. WHO's work to improve the mental health of individuals and society at large includes the promotion of mental health well‐being, prevention of mental disorders, protection of human rights and caring for people affected with mental health conditions.

#### 1.2.2 Key to ob**t**ain sound mental health

The only sustainable method for reducing the mental health burden is through prevention of mental illness and promotion of mental health. Mental health is fundamental to good health and well‐being and influences social and economic outcomes across the lifespan (Barry & Friedli, 2008; Durlak & Wells, 1997; Jenkins et al., 2011). This is possible with understanding of effective mental health and social interventions available and to enhance further research in the areas undone to raise the quality of community health services reaching the population.

Systematic reviews (SRs) of the international evidence, which come predominantly from HICs, show that comprehensive mental health promotion interventions carried out in collaboration with families, schools and communities, lead to improvements not only in mental health but also improved social functioning, academic and work performance, and general health behaviours (Barry et al., 2019; Baker‐Henningham & López Bóo, 2010; Herrman et al., 2005; Jané‐Llopis et al., 2005; Nores & Barnett, 2010; Stewart‐Brown & Schrader‐Mcmillan, 2011; Weare & Nind, 2011).

The objective of this evidence and gap map (EGM) is to identify and map the interventions on mental health globally and identify evident gaps in evidence segregated by important intervention categories, region, context and population subgroup.

**Table 1:** List of intervention and subintervention categories

Interventions	Subintervention categories
Governance and leadership	o Policies and law (legal rights and access to justice) o Positions in public institutions and Judiciary o Active surveillance systems
Community based‐mental health services	o Human resource development o Community mobilisation o Social media and m‐health o Home‐visits o Facility‐based interventions
Promotion	o Access to mental health services o Self –help groups/community networks o Social skills training o Social protection
Preventive	o Rehabilitation o Educational interventions o Workplace interventions (Supported employment)

*The intervention categories are: Governance and leadership, Community based‐mental health services, promotion and preventive interventions*.

### 1.3 How the intervention might work

The proposed EGM will be unique as this will be the first global EGM on Mental Health interventions. It will be based on World Health Organisation's Comprehensive mental health action plan 2013–2020. Intended users can be researchers, helping them identify the available literature in this field and to explore the findings and the quality of existing evidence and facilitate informed judgement.

### 1.4 Why it is important to do this review

There is an ongoing Campbell EGM on disability in LMICs. Also, there is an existing map on acupuncture for mental health by Department of Veteran Affairs. However, the former map focuses only on LMICs and the later focusses on specific interventions only. There are few SRs available on effectiveness of interventions for the promotion of mental health among the young people in LMICS (Barry et al. 2013; Das et al. 2016).

## 2 OBJECTIVES

*The specific objectives of this map are to:*

I. Develop a clear framework of types of interventions and outcomes to provide an overview of available evidence on the interventions available to improve the well‐being of people with mental disorders.
II. Map available SRs and primary studies on the effectiveness of interventions in this framework, with an overview provided in a summary report.
III. Provide database entries of included studies which summarise the intervention, context, study design and main findings.

## 3 METHODS

### 3.1 Criteria for considering studies for this review

#### 3.1.1 Types of studies

The EGM will include SRs of effects of interventions and effectiveness studies that used either: (a) randomised experimental design, or (b) rigorous quasi‐experimental design, (c) natural experiments, (d) regression discontinuity, (e) propensity score matching, (f) difference in difference, (g) instrumental variables, (h) and other matching design, (I) Single subject design. We will include qualitative studies, relevant working papers will also be included.

#### 3.1.2 Types of participants

1. **Population:** The target populations are people with mental disorders. Mental disorders comprise a broad range of problems, with different symptoms. However, they are generally characterised by some combination of abnormal thoughts, emotions, behaviour and relationships with others. Examples are schizophrenia, depression, intellectual disabilities and disorders due to drug abuse. Population subgroups include: Children (under 14 years of age), young adulthood (15–24), middle adulthood (25–44), and older adulthood (45–64) and Elderly 65+ years old.
2. **Context:** Ethnic minorities, people in conflict affected regions, women, men, low income families, unemployed, homeless, victims of sexual and physical abuse, refugees.
3. **Region:** World Bank regions: East Asia and Pacific, Europe and Central Asia, Latin America and Caribbean, Middle East and North Africa, North America, South Asia, Sub‐Saharan Africa.
4. **WHO income groups:** Low, lower‐middle, upper‐middle, and high based on the World Bank list of analytical income classification of economies for the fiscal year, which is based on the Atlas gross national income per capita estimates (Annex: 1 and 2).

#### 3.1.3 Types of interventions

##### Governance and leadership

Governance in the health sector refers to a wide range of steering and rule‐making related functions carried out by governments/decisions makers as they seek to achieve national health policy objectives that are conducive to universal health coverage. Governance is a political process that involves balancing competing influences and demands. A strong civil leadership in the community and organisations for people experiencing mental health conditions and psychosocial disabilities can be of great help to enforce effective and accountable policies, laws and services in a manner consistent with international as well as regional human rights associations including their caregivers and close family members as well.

- *Policies and law (legal rights and access to justice)*
  Policies are a set of statements or commitments to pursue courses of action aimed at achieving defined goals of public or private institutions (EURO, 1999) given by WHO
  Mental health laws that are independent of legislative documentation or integration into other health and capacity‐related laws involving key principles, values and objectives of policy for mental health by establishing legal and oversight mechanisms to promote human rights and the development of accessible health and social services in the community.
- *Positions in public institutions and Judiciary*
  Education, employment, disability, judicial system, human rights protection, social protection, poverty reduction and development are important means of meeting the multidimensional requirements of mental health systems. Hence Motivating and engaging the stakeholders from all relevant sectors, including persons with mental disorders, carers and family members, in developing and implementing policies, laws and services related to mental health, through a formalised mechanism.
- *Active surveillance systems*
  Good‐quality mental health service systems that are available including social interventions for mentally disordered people and the interventions/initiatives from health workers in improving well‐being of people with mental disorders and vice versa, because of the high rates of co‐morbid mental health problems.

### 3.2 Community based‐mental health services

Community‐based service delivery for mental health needs to encompass a recovery‐based approach that puts the emphasis on supporting individuals with mental disorders and psychosocial disabilities to achieve their own aspirations and goals. A multi sectoral approach in providing supportive services to the individuals, at different stages of the life course and, as appropriate, facilitate their access to human rights such as employment (including return‐to‐work programmes), housing and educational opportunities, and participation in community activities, programmes and meaningful activities.

- *Human resource development*
  Knowing skills of healthcare providers in delivering evidence‐based care, culturally appropriate and human rights‐oriented mental health and social care services for the mentally disordered people by introducing mental health into undergraduate and graduate curricula. Available training and mentoring services of health workers in the field, particularly in nonspecialised settings, to identify people with mental disorders receiving appropriate treatment and support as well as to identifying affected people and referring them to higher levels of care.
- *Community mobilisation*
  People with mental disorders often live in vulnerable situations and may be excluded and marginalised from society, which constitutes a significant impediment to the achievement of national and international development goals. The Convention on the Rights of Persons with Disabilities, which is binding on States Parties that have ratified or acceded to it, protects and promotes the rights of all persons with disabilities, including persons with mental and intellectual impairments, and also promotes their full inclusion in international cooperation including international development programmes. (Comprehensive Mental health action plan 2013–2020).
- *Social media and m‐health*
  Not only does service integration require the acquisition of new knowledge and skills to identify, manage and refer people with mental disorders as appropriate, but also the redefinition of health workers' roles and changes to the existing service culture and attitudes of general health workers, social workers, occupational therapists and other professional groups. Furthermore, in this context, the role of specialised mental health professionals needs to be expanded to encompass supervision and support of general health workers in providing mental health interventions (Xiong & Phillips, 2016).
- *Home‐visits*
  Interim HealthCare support can help individuals with mental disorders along with their family support. Interim's qualified psychiatric nurses, counsellors and social workers work along with mental health professionals including psychiatrists and psychologists creating a home care plan supporting an individual's treatment and management of their daily life. Mental Health Home Care and Interim HealthCare (2019).
- *Facility‐based interventions*
  Interventions like improving physical activities and counselling or other various facilities available precisely for the people with mental disorders will be mapped.

### 3.3 Mental health Promotion

WHO defines health promotion as “the process of enabling people to increase control over, and to improve their health” (WHO, 2004). Mental health promotion often refers to positive mental health, rather than mental ill health. Positive mental health is the desired outcome of health promotion interventions. Promotion is about improving health and well‐being of the individuals. By identifying the positive aspects of mental health. The health promotional activities like improved access to mental health services and forming self‐help groups through small community networks and conducting promotive mental health programmes in early identification of mental disorders through accurate screening modalities. Improving social protection through social skills training activities to improve verbal/Nonverbal skills, social perception, assertiveness, conversational skills, expressions, and management and stabilisation of one's illness depending on the individual's or family's needs and requirements are some of the interventions that promote the mental health of the affected individuals.

### 3.4 Mental illness prevention

Mental ill‐health refers to mental health problems, symptoms and disorders, including mental health strain and symptoms related to temporary or persistent distress. Preventive interventions work by focusing on reducing risk factors and enhancing protective factors associated with mental ill‐health (WHO, 2004), whereas, Public health definition of mental disorder prevention. Mental disorder prevention aims at “reducing incidence, prevalence, recurrence of mental disorders, the time spent with symptoms, or the risk condition for a mental illness, preventing or delaying recurrences and also decreasing the impact of illness in the affected person, their families and the society” (Haggerty & Mrazek, 1994) Preventing mental disorders is concerned with avoiding disease through following interventions;

- *Rehabilitation*
  Psychiatric rehabilitation is to help individuals with persistent and serious mental illness to develop the emotional, social and intellectual skills needed to live, learn and work in the community with the least amount of professional support (Mental Health Home Care and Interim HealthCare, 2019)
- *Educational interventions*
  To identify the educational interventions promoted in schools for children and adolescents with mental disorders or early educational interventions in schools for preventing the occurrence of any mental health disorders in future.
- *Workplace interventions (supported employment)*
  Organisational level interventions designed to include training in an individually tailored focus such as learning coping skills. Without such training enhanced organisational benefits such as opportunities for more job control or increased participation may not be utilised by employees (Mental Health Home Care | Interim HealthCare, 2019).

#### 3.4.1 Types of outcome measures

Primary outcome measures are detailed below

##### Primary outcomes

A community‐based rehabilitation (CBR) programme is formed by one or more activities in one or more of the five components (health, education, livelihood, social and empowerment) (Khasnabis et al., 2010). A part of CBR matrix for the social outcomes has been modified with the addition of Quality of Life as an outcome that has been adapted from the World Health Organisation's comprehensive mental health action plan (2013–2020).

###### Health

Training People with compromised mental health/poor cognitive development and their family members or their immediate caregivers in locating the social interventions for mental health activities like screening for mental health conditions and impairments, providing counselling and information regarding rehabilitation facilities for the people with mental disorders and gathering information on health status as listed below on;

- Morbidity and mortality
- Mental health and cognitive development
- Access to mental health services

###### Education

Providing education and training for families or caregivers of mentally disordered people and making services accessible to them through,

- Access to educational services
- Enrolment/attendance
- Life and social skills

###### Quality of life

Quality of Life as an individual's perception of their position in life in the context of the culture and value systems in which they live and in relation to their goals, expectations, standards and concerns. It is a broad ranging concept affected in a complex way by the person's physical health, psychological state, personal beliefs, social relationships and their relationship to salient features of their environment.

- Physical health
- Psychological
- Level of independence
- Environment
- Spirituality/religion/personal beliefs

###### Livelihood

Linking the jobseeker with mental disorder to existing support services; advocating before relevant public and private agencies to ensure accessible supportive programmes and services from the government for their better livelihood, may be specified to following;

- Access to job market
- Access to financial services
- Access to social protection programme
- Poverty and out‐of‐pocket payment
- Control over own money

###### Social outcomes

Converting treating and teaching institutions as specified rehabilitation/counselling centres for the mentally disordered people; providing information about the opportunities/activities that are available within the community for improving social outcomes like;

- Prosocial, leisure and relationship
- Social functioning
- Stigma and discrimination
- Safety

###### Empowerment

Helping people with mental disorders involving community organisations in CBR planning, implementation, and monitoring;

- Informed choices
- Representation and community level
- Advocacy
- Voting rights

(ANNEX 3)

### 3.5 Search methods for identification of studies

Studies in English language and from any country will be reviewed for inclusion.

Electronic Search will be conducted with the help of a relevant databases in our area of study. Key words with synonyms will be used for the specific search to be conducted. It is planned to search the following databases/search engines (Refer to Annex: 2).

**International organisations**

− ILO
− WHO
− 3ie evidence and gap map repository
− Cochrane
− EPPI Centre Evaluation Database of Education Research

#### 3.5.1 Electronic searches

##### Academic databases

− PubMED
− Econlit
− The National Bureau of Economic Research (NBER)
− Social Science Research Network (SSRN)
− International Bibliography of Social Sciences (IBSS)
− CINHAL
− SCOPUS
− Social Sciences Citation Index (Web of Science)
− ‐Psyc INFO
− ‐Research for development

##### SR database

− Cochrane library
− Campbell library
− 3ie Systematic Review Database

#### 3.5.2 Searching other resources

Grey literature.

### 3.6 Data collection and analysis

There will be four independent reviewers working in this EGM on EPPI reviewer 4. Each one of them will be responsible to carry out the search and screening. Independent title and abstract screening will be done and the selected studies will be included for the next stage of full text review after discussion with the team members. In case of any doubts, consensus from the third person will be taken and then decided on exclusion of the paper. The final consensus will be taken with the subject experts. The latter will follow the same for full text screening.

#### 3.6.1 Selection of studies

Title and abstracts will be reviewed initially by four independent authors in Eppi reviewer‐4 and any studies will be checked for duplication. Excluded studies will be reported after an expert suggestion. Following this, full text screening of selected studies from the title abstract and stage will be conducted in relation to the set inclusion/exclusion criteria's. If there is a considerable level of disagreement, the third reviewers will resolve the conflict. The study will be assessed for quality using AMSTAR‐2 scale. Any potential differences in interpretation will be discussed and resolved by the expert team.

#### 3.6.2 Data extraction and management

We will code each included study using a piloted coding tool covering study characteristics, population, intervention and outcomes (ANNEX 3).

#### 3.6.3 Assessment of risk of bias in included studies

AMSTAR‐2 for SRs and modified risk of bias for primary studies.

#### 3.6.4 Dealing with missing data

The lead author will be contacted for retrieval of the missing data. If the author would fail to respond back then we will exclude the article from the review reporting it as Missing data.

#### 3.6.5 Assessment of heterogeneity

Not planned for now.

#### 3.6.6 Data synthesis

If it is possible to conduct a meta‐analysis with the available data, Review Manager Software from the Cochrane Collaboration will be used to analyse the data. If statistical pooling is not possible the findings will be presented in narrative form. The data will be mapped as intervention and outcome tables.

#### 3.6.7 Subgroup analysis and investigation of heterogeneity

Subgroup analysis will be conducted to assess the heterogeneity between the included studies.

#### 3.6.8 Sensitivity analysis

Sensitivity analysis will be performed to assess the impact of methodological quality. Analysis will be conducted by excluding the included studies at high risk of bias for any one or more of selection, attrition, or detection bias. The Meta‐analysis will be repeated after removing the lower quality studies.

#### 3.6.9 Summary of findings and assessment of the certainty of the evidence

AMSTAR‐2 will be used for assessment of SRs

## CONTRIBUTIONS OF AUTHORS

**Table:** 

Authors	Contribution				
	TRF		Protocol		
	Concept initiation	Writing	Writing	Coding	Search strategy
Sherize Dsouza	*	*	*	*	*
Jisha B. Krishnan	*	*	*	‐	‐
Ashrita Saran	*	*	*	*	*

## DECLARATIONS OF INTEREST

There is no conflict of interest.

## APPENDIX A

### Population coding variables

**Table:**

**Population**	**Subgroups**	**Coding variables**
	Children	≤14 years of age
	Young adulthood	≥15 to ≤24
	Middle adulthood	≥25 to ≤44
	Older adulthood	≥45 to ≤64
	Elderly	≥65

### APPENDIX B: Intervention coding variables

**Table:**

Category		Answer
Descriptive information	Title	Open answer
	Author citation	Open answer
	Publication Date	Open answer
	URL	Open answer
	Volume no	Open answer
	Issue no	Open answer
Geographical information	World Bank region	− South Asia − Sub‐Saharan Africa − East Asia and Pacific − Europe and Central Asia − Latin America and Caribbean − Middle East and North Africa − North America
	Country	− Low income − Lower Middle income − Upper Middle income − High income − Conflict affected (to see relevant country list as per World Bank Region)
Study design		− Systematic reviews − Overviews − RCT − Quasi‐experimental study − Case‐control − Cohort − Controlled trial
Population		− Children − Adults − Elderly − Women − Men − LGBT community − Conflict affected − Disadvantaged − Migrants/refugees − Ethnic minorities
Type of impairment		− Mental retardation − Mental distress − Intellectual/learning impairment
Intervention		− Governance and Leadership Policies and Law (legal rights and access to Justice) Positions in Public Institutions and Judiciary Active surveillance systems
		Community Based ‐ Mental Health Services
		Human Resource development community mobilisation Social media and m‐health Home visits Facility based interventions − Promotion Access to mental health services Self‐help groups/community networks social skills training Social protection − Preventive Rehabilitation Educational interventions Workplace interventions (Supported employment)

### APPENDIX C: Outcome coding variables

**Table:**

Outcome	Health
	Morbidity and mortality
	Mental health and cognitive development
	Access to mental health services
	− Education
	Access to educational services
	Enrolment/Attendance (Enrolment to primary, secondary and tertiary education)
	Life and social skills
	− Quality of Life
	Physical Health
	Psychological
	Level of Independence
	Environment
	Spirituality/Religion/Personal beliefs
	− Social
	Pro social, leisure and relationship
	Social functioning
	Stigma and discrimination
	Safety
	− Livelihood
	Access to job market
	Access to financial services
	Access to social protection programme
	Poverty and out‐of‐pocket payment
	Control over own money
	− Empowerment
	Informed choices
	Representation at community level
	Advocacy
	Voting rights
